# Anticholinergic Burden and Falls in Older Adults: The Role of Functional Status

**DOI:** 10.3390/jcm15207811

**Published:** 2026-10-10

**Authors:** Feyza Mutlay, Murat Das

**Affiliations:** 1 Division of Geriatrics, Department of Internal Medicine, Faculty of Medicine, Canakkale Onsekiz Mart University, Canakkale 17000, Türkiye; 2 Department of Emergency Medicine, Faculty of Medicine, Canakkale Onsekiz Mart University, Canakkale 17000, Türkiye; muratdas58@gmail.com

**Keywords:** falls, anticholinergic burden, functional status, activities of daily living, older adults, geriatric assessment

## Abstract

**Background:** Falls are a major geriatric problem associated with functional decline, loss of independence, and increased healthcare utilization. Although anticholinergic burden and impaired functional status are recognized risk factors, their combined association with recurrent falls remains unclear. This study evaluated how anticholinergic burden and functional status relate to recurrent falls in older adults. **Methods:** This retrospective cross-sectional study included 347 patients aged ≥65 years who underwent comprehensive geriatric assessment. Anticholinergic burden was assessed with the Anticholinergic Cognitive Burden (ACB) score, functional status with the Barthel Index and Lawton Instrumental Activities of Daily Living (IADL) Scale, and comorbidity with the Charlson Comorbidity Index. Univariable and multivariable logistic regression and receiver operating characteristic analyses were performed; the interaction between ACB ≥ 2 and functional impairment was also tested. **Results:** Recurrent falls were reported by 108 patients (31.1%). Patients with recurrent falls were older, had higher medication use, ACB scores, and comorbidity burden, and lower Barthel Index and Lawton IADL scores (all *p* < 0.05). In univariable analyses, ACB scores of 2 and 3 were associated with increased odds of recurrent falls. In multivariable analyses, both the Barthel Index (aOR = 0.96, 95% CI: 0.94–0.98; *p* < 0.05) and Lawton IADL score (aOR = 0.91, 95% CI: 0.87–0.96; *p* < 0.05) remained independently associated with recurrent falls. The Barthel Index and Lawton IADL Scale showed the highest discriminatory performance (AUC = 0.75 and 0.74, respectively). Patients with Lawton IADL ≤ 19 and ACB ≥ 2 had the strongest combined association with recurrent falls (OR = 12.04, 95% CI: 5.88–24.66; *p* < 0.05). This association remained significant after multivariable adjustment for age, sex, number of medications, and comorbidity burden (aOR = 7.93, 95% CI: 3.51–17.90; *p* < 0.001). The interaction between ACB ≥ 2 and functional impairment was not statistically significant (*p* for interaction = 0.271 for the Barthel Index and 0.285 for the Lawton IADL Scale); therefore, the findings are described as joint (combined) associations rather than effect modification. **Conclusions:** Functional status remained independently associated with recurrent falls after adjustment for age, sex, anticholinergic burden, medication count, and comorbidity burden. In this cross-sectional study, functional impairment and higher anticholinergic burden showed a joint association with recurrent falls, without evidence of statistical interaction; longitudinal studies are needed to clarify temporal and causal relationships.

## 1. Introduction

Falls in older adults are among the most significant geriatric syndromes because of their association with fractures, hospitalization, loss of functional independence, institutional care needs, and mortality [1]. Approximately one-third of individuals aged 65 and older in the general population experience at least one fall each year, and this rate rises with advancing age and increasing frailty [2]. Falls are multifactorial events resulting from the interaction of numerous intrinsic and extrinsic risk factors, including balance and gait disorders, declines in cognitive and visual function, functional dependence, environmental factors, and medication use [1]. Among these risk factors, medication use is considered a fundamental component of fall prevention strategies because it is modifiable through appropriate medication review and deprescribing approaches [1,3].

Anticholinergic medications are widely used to treat many clinical conditions common in older adults, including depression, urinary incontinence, chronic respiratory diseases, gastrointestinal disorders, allergic conditions, and neurological diseases [4]. Exposure to anticholinergic drugs is common among older adults living in the community, with studies reporting rates of approximately 40–60% [5]. In older adults, the frequent use of multiple anticholinergic drugs together has led to the clinically significant concept of anticholinergic cognitive burden (ACB), which represents total anticholinergic exposure rather than individual drug exposure. ACB scale, one of the most commonly used tools for assessing anticholinergic burden, was developed to rate the anticholinergic properties of drugs and is widely used in both clinical practice and research [6,7].

Due to age-related pharmacokinetic and pharmacodynamic changes, older adults are more susceptible to the adverse effects of anticholinergic drugs. Physiological changes, including decreased renal and hepatic function, increased blood–brain barrier permeability, and reduced central cholinergic reserve, exacerbate the central and peripheral side effects of these medications [4,8]. Anticholinergic drugs may increase the risk of falls through mechanisms such as sedation, dizziness, blurred vision, cognitive impairment, decreased attention, orthostatic hypotension, and impaired gait and balance [3,9]. Additionally, some psychotropic drugs, such as benzodiazepines, antipsychotics, and antidepressants, have been associated with an increased risk of falls in older adults due to their sedative and cognitive effects, and some agents may contribute to the anticholinergic burden [10].

Observational studies and systematic reviews generally show a positive association between high anticholinergic burden and fall risk [11]. However, substantial heterogeneity exists across studies in anticholinergic burden scales, fall definitions, and statistical approaches. Importantly, the role of functional status in the relationship between anticholinergic burden and falls remains poorly understood. This study aims to evaluate the relationship among anticholinergic burden, functional status, and fall history in older adult individuals presenting to a geriatrics outpatient clinic.

## 2. Materials and Methods

### 2.1. Study Design and Participants

This retrospective cross-sectional study was conducted among older adults admitted to the geriatric outpatient clinic between May 2025 and March 2026. Patients aged ≥65 years who underwent comprehensive geriatric assessment were eligible for inclusion.

**Figure 1:**
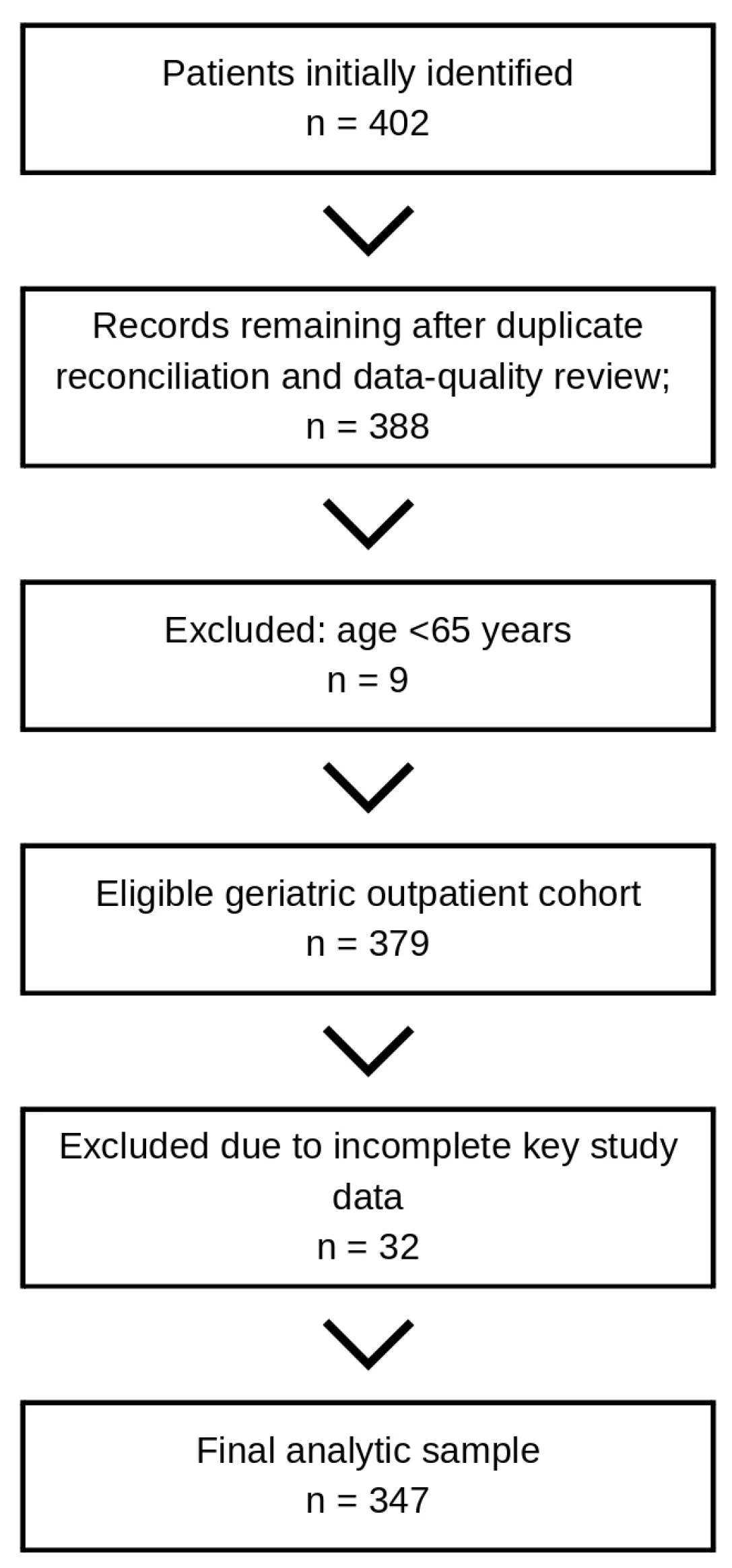
Flow diagram of patient selection and inclusion in the final analytic sample.

Demographic characteristics, clinical data, medication profiles, laboratory findings, and functional assessment results were obtained from electronic medical records. Patients with missing data on the primary study variables, including anticholinergic burden, functional status, comorbidity burden, or fall history, were excluded from the analysis. Patients who met the predefined inclusion and exclusion criteria and underwent comprehensive geriatric assessment were included in the final analysis, regardless of their fall status, resulting in a total of 347 patients (Figure 1).

The study protocol was approved by the local ethics committee (Canakkale Onsekiz Mart University Non-Interventional Research Ethics Committee, dated 11 March 2026, decision no. 2026-04/04-03). The study was conducted in accordance with the Declaration of Helsinki.

### 2.2. Fall Assessment

Fall history was assessed using information from the comprehensive geriatric assessment and recorded in the electronic medical records. As part of the comprehensive geriatric assessment, all patients attending the geriatric outpatient clinic routinely completed a structured questionnaire that included questions about geriatric syndromes and recurrent falls over the previous 12 months. For the present retrospective study, fall history was identified from these routinely recorded assessment data and was not based on a specific diagnostic or coding system. A fall was defined as an event in which an individual unintentionally came to rest on the ground, floor, or a lower level without overwhelming external force. Recurrent falls were defined as two or more falls within the previous 12 months [1].

Patients were categorized into two groups based on the presence or absence of recurrent falls during the previous 12 months.

### 2.3. Anticholinergic Burden Assessment

Anticholinergic burden was assessed using the Anticholinergic Cognitive Burden (ACB) scale, based on the original scale described by Boustani et al. [6] and the subsequent 2012 update [12]. On this scale, medications are scored according to their anticholinergic activity. Medications with no anticholinergic activity were assigned a score of 0; those with possible anticholinergic effects were assigned a score of 1; and those with clinically relevant anticholinergic effects were assigned a score of 2 or 3, according to the ACB scale and its 2012 update [12]. The total ACB score represents the sum of the ACB ratings of all regularly used medications and therefore does not correspond to the number of anticholinergic medications. For example, a total ACB score of 2 may result from one medication rated 2 or from two medications each rated 1. Individual medications are assigned ACB scores ranging from 1 to 3 according to their anticholinergic activity. Because the total score is calculated by summing the scores of all relevant medications, there is no fixed theoretical maximum total ACB score; however, in the present analysis the total ACB score was top-coded at 3, i.e., the category labelled “3” represents a total ACB score of ≥3 (observed range 0–3). The assessment was based on regularly used medications at the time of the comprehensive geriatric assessment, while intermittently used or as-needed (PRN) medications were excluded. Medication dose and duration of exposure were not taken into account in the ACB calculation. Medication exposure, fall history, and functional status were assessed during the same comprehensive geriatric assessment period.

For statistical analyses, anticholinergic burden was treated as a categorical variable. For logistic regression analyses, patients were categorized by total ACB score as follows: ACB score = 0 (no anticholinergic exposure), ACB score = 1, ACB score = 2, and ACB score = 3 (top-coded; i.e., ≥3), with ACB score = 0 used as the reference category. For receiver operating characteristic (ROC) analysis, an ACB score ≥ 2 was evaluated as a cutoff for discriminating patients with and without a history of recurrent falls. This cutoff was subsequently used in the combined-category models with functional status measures. These categories were used to evaluate the association between anticholinergic burden and history of recurrent falls.

### 2.4. Medication Assessment

Regularly used medications were obtained from electronic medical records from the comprehensive geriatric assessment. Regularly used medications were defined as those routinely taken per the patient’s established medication regimen at the time of assessment. Intermittently used or as-needed (PRN) medications were excluded. All regularly used medications were recorded, regardless of anticholinergic properties. The total number of medications was recorded, and polypharmacy was defined as the use of five or more medications [13].

Psychotropic medication use was also evaluated among regularly used medications. Selective serotonin reuptake inhibitors (SSRIs), serotonin–norepinephrine reuptake inhibitors (SNRIs), antipsychotics, benzodiazepines, and opioids were identified and recorded as separate medication categories. These medications were included in the total medication count and were also evaluated as specific medication categories.

### 2.5. Functional Status Assessment

Functional status was evaluated as part of the comprehensive geriatric assessment using standardized instruments. Basic activities of daily living (BADL) were assessed with the Barthel Index (score range: 0–100), which evaluates feeding, bathing, grooming, dressing, bowel and bladder control, toileting, transfers, mobility, and stair use [14]. Instrumental activities of daily living (IADL) were evaluated using the Lawton Instrumental Activities of Daily Living Scale [15], with the 23-point polytomous scoring method (score range: 0–23) described in a previous methodological study [16] and used in a Turkish older-adult population [17]. A Turkish version of the Lawton IADL has been evaluated for validity and reliability [18]. For both instruments, higher scores indicate better functional status, with the maximum score representing the highest level of functional independence for each scale. The scores were analyzed within their respective scoring ranges and were not directly compared using their absolute values.

### 2.6. Assessment of Comorbidity and Clinical Variables

Comorbidity burden was assessed using the Charlson Comorbidity Index (CCI) [19], a weighted index that summarizes the burden of predefined comorbid conditions. Demographic characteristics included age and sex. Clinical variables included systolic and diastolic blood pressure and the presence of dementia, diabetes mellitus, hypertension, cardiovascular disease, chronic kidney disease, and prior stroke. Laboratory parameters included hemoglobin, vitamin D, vitamin B12, and thyroid-stimulating hormone (TSH) levels. These variables were obtained from electronic medical records and the comprehensive geriatric assessment.

### 2.7. Statistical Analysis

Statistical analyses were conducted using IBM SPSS Statistics version 26. 0 (IBM Corp., Armonk, NY, USA). Continuous variables were reported as the median and interquartile range (IQR), and categorical variables as counts and percentages. Comparisons between patients with and without recurrent falls were assessed using the Mann–Whitney U test for continuous variables and the Pearson chi-square test or Fisher’s exact test, as appropriate, for categorical variables.

Univariable binary logistic regression was used to evaluate factors associated with recurrent falls. Recurrent falls (≥2 falls) were defined as the dependent variable, with participants reporting no falls or a single fall serving as the reference group. Results were reported as odds ratios (ORs) with 95% confidence intervals (CIs) and corresponding *p* values. For categorical variables with more than two levels, the clinically lowest-risk category was used as the reference category. To identify factors independently associated with recurrent falls, two multivariable binary logistic regression models were constructed. Because the Barthel Index and Lawton IADL Scale assess related dimensions of functional status, they were entered into separate models. Model 1 included age, sex, ACB score category, number of medications, Charlson Comorbidity Index, and Barthel Index. Model 2 included the same covariates, with the Lawton IADL score replacing the Barthel Index. ACB score 0 was used as the reference category. Adjusted odds ratios (aORs) with 95% confidence intervals were reported.

Receiver operating characteristic (ROC) curve analyses were conducted to assess the discriminative ability of the Barthel Index, Lawton Instrumental Activities of Daily Living (IADL) Scale, Anticholinergic Cognitive Burden (ACB) score, and Charlson Comorbidity Index (CCI) in identifying patients with recurrent falls. The area under the ROC curve (AUC) was computed for each score. Optimal cutoff values were determined by maximizing the Youden index (J = sensitivity + specificity − 1). At these cutoffs, sensitivity, specificity, positive predictive value (PPV), negative predictive value (NPV), and overall accuracy were calculated. Ninety-five percent confidence intervals for these performance measures were estimated using the Wilson method.

To assess the association of combined clinical characteristics with fall history, four-level categorical variables were constructed by combining dichotomized functional status measures with the CCI or ACB score, using Youden-based cutoffs. The cutoffs were ≤90 for the Barthel Index, ≤19 for the Lawton IADL Scale, ≥2 for the ACB score, and ≥3 for the CCI. For each combined model, the category representing preserved functional status, together with the lower-risk CCI or ACB category, served as the reference group. Associations between each combined category and fall history were evaluated using binary logistic regression and reported as ORs with 95% CIs. In addition, each combined category variable was entered into a multivariable binary logistic regression model to determine whether the observed associations persisted after adjustment for potential confounders. All combined models were adjusted for age, sex, and number of medications. Models containing a CCI-based combined variable were additionally adjusted for ACB score category (0, 1, 2, and 3, with ACB score 0 as the reference category), whereas models containing an ACB-based combined variable were additionally adjusted for the CCI. The two component variables forming each combined category were not entered separately into the corresponding model in order to avoid collinearity with the combined variable. Adjusted odds ratios (aORs) with 95% CIs were reported, and the overall effect of each combined category variable was evaluated using the Wald chi-square test. To formally test whether functional impairment modified the association between anticholinergic burden and fall history, a multiplicative interaction term (ACB ≥ 2 × functional impairment) was added to the adjusted ACB-based models (Barthel Index–ACB and Lawton IADL–ACB). In these models, the two dichotomized component variables were entered together with their product term, adjusted for age, sex, number of medications, and CCI, and the *p* value of the interaction term (Wald test) was reported. If the interaction was not significant, the findings were interpreted as a joint (combined) association rather than effect modification.

All statistical tests were two-sided, and a *p*-value < 0.05 was considered statistically significant.

## 3. Results

### 3.1. Baseline Characteristics

A total of 347 older adults were included in the analysis, of whom 108 (31.1%) had a history of recurrent falls in the previous 12 months. Patients with recurrent falls were older than those without such a history [median age, 78.5 (IQR: 74.0–84.0) vs. 74.0 (IQR: 70.0–79.0) years; *p* < 0.05]. They also had a higher number of medications [7.0 (5.0–10.0) vs. 5.0 (3.0–7.5); *p* < 0.05] and a higher prevalence of polypharmacy (76.9% vs. 58.2%; *p* < 0.05).

The distribution of ACB scores differed significantly between groups (*p* < 0.05). Compared with patients without recurrent falls, those with recurrent falls had a lower proportion with an ACB score of 0 and higher proportions with ACB scores of 2 and 3. Patients with recurrent falls also had a higher comorbidity burden, reflected by higher CCI values [2.0 (1.0–4.0) vs. 1.0 (0.0–2.0); *p* < 0.05]. Dementia (29.6% vs. 5.9%; *p* < 0.05), cardiovascular disease (36.1% vs. 24.3%; *p* = 0.023), and chronic kidney disease (47.2% vs. 20.1%; *p* < 0.05) were more frequent among patients with recurrent falls.

Functional status differed markedly between groups. Patients with recurrent falls had significantly lower Barthel Index scores [85.0 (72.5–95.0) vs. 100.0 (90.0–100.0); *p* < 0.05] and Lawton IADL scores [16.0 (8.0–21.0) vs. 22.0 (19.0–23.0); *p* < 0.05], indicating greater functional impairment. In addition, systolic and diastolic blood pressure and hemoglobin levels were lower in patients with recurrent falls (Table 1).

### 3.2. Medication Use and Anticholinergic Burden

Psychotropic medication use was more frequent among patients with recurrent falls (50.0% vs. 28.5%; *p* < 0.05). Among individual medication classes, SSRI use (32.4% vs. 20.1%; *p* < 0.05) and antipsychotic use (19.4% vs. 4.6%; *p* < 0.05) were significantly more common in patients with recurrent falls, whereas no significant differences were observed for SNRI, benzodiazepine, or opioid use (Table 2).

### 3.3. Independent Factors Associated with Recurrent Falls

In univariable logistic regression, increasing age, higher medication burden, polypharmacy, higher CCI, dementia, cardiovascular disease, chronic kidney disease, and psychotropic medication use were associated with recurrent falls. Higher functional scores were inversely associated with recurrent falls; each one-point increase in the Barthel Index (OR = 0.93; 95% CI: 0.91–0.95; *p* < 0.05) and the Lawton IADL score (OR = 0.87; 95% CI: 0.83–0.90; *p* < 0.05) was associated with lower odds of recurrent falls.

Regarding anticholinergic burden, an ACB score of 1 was not associated with recurrent falls compared with an ACB score of 0 (OR = 0.85; 95% CI: 0.43–1.67; *p* > 0.05). In contrast, ACB scores of 2 and 3 were associated with higher odds of recurrent falls (OR = 5.00; 95% CI: 2.16–11.59; *p* < 0.001 and OR = 3.07; 95% CI: 1.58–5.96; *p* < 0.05, respectively) (Table 3).

### 3.4. Discriminative Performance of Functional and Anticholinergic Measures

ROC curve analyses showed that functional status measures had the highest discriminatory performance for identifying patients with recurrent falls. The Barthel Index had an AUC of 0.75, with an optimal cutoff of ≤90, while the Lawton IADL Scale had an AUC of 0.74, with an optimal cutoff of ≤19. The Lawton IADL Scale had the highest Youden index (J = 0.40). The ACB score had lower discriminatory performance (AUC = 0.61), although an ACB cutoff of ≥2 had relatively high specificity (86.6%). The CCI had an AUC of 0.64, with an optimal cutoff of ≥3 (Table 4). These analyses reflect the cross-sectional discrimination of a history of recurrent falls rather than prediction of future falls, and the cutoffs were derived from the same sample.

### 3.5. Combined Association of Functional Impairment and Anticholinergic Burden

Combined-category analyses estimated the joint association of functional status and comorbidity or anticholinergic burden with fall history. In the Barthel Index–CCI model, patients with impaired functional status (Barthel Index ≤ 90) and a higher comorbidity burden (CCI ≥ 3) had the highest prevalence of fall history (60.3%) and 8.9-fold higher odds than the reference group (OR = 8.87; 95% CI: 4.59–17.16; *p* < 0.05).

Similarly, in the Barthel Index–ACB model, the combination of impaired Barthel Index and elevated ACB score was associated with increased odds of fall history (OR = 9.91; 95% CI: 4.85–20.25; *p* < 0.05). The strongest association was observed in the Lawton IADL–ACB model. Although an ACB score ≥ 2 alone among patients with preserved IADL function was not significantly associated with fall history (OR = 1.51; 95% CI: 0.52–4.42; *p* > 0.05), patients with both Lawton IADL impairment (≤19) and elevated ACB score (≥2) had a 70.0% prevalence of fall history and approximately 12-fold higher odds than patients with preserved IADL function and lower ACB scores (OR = 12.04; 95% CI: 5.88–24.66; *p* < 0.05) (Table 5).

These associations persisted after multivariable adjustment. In the Barthel Index–CCI model, adjusted for age, sex, number of medications, and ACB score category, patients with a Barthel Index ≤ 90 and a CCI ≥ 3 remained at substantially higher odds of recurrent falls (aOR = 5.01; 95% CI: 2.28–11.01; *p* < 0.05). In the Barthel Index–ACB model, adjusted for age, sex, number of medications, and CCI, the combination of an impaired Barthel Index and an elevated ACB score also remained independently associated with recurrent falls (aOR = 6.15; 95% CI: 2.75–13.77; *p* < 0.05). Similarly, in the Lawton IADL–CCI model, patients with a Lawton IADL score ≤ 19 and a CCI ≥ 3 had higher adjusted odds of recurrent falls (aOR = 5.71; 95% CI: 2.57–12.66; *p* < 0.05). The strongest adjusted association was again observed in the Lawton IADL–ACB model, in which patients with both IADL impairment (Lawton IADL ≤ 19) and an elevated ACB score (≥2) had approximately eight-fold higher adjusted odds of recurrent falls than patients with preserved IADL function and lower anticholinergic burden (aOR = 7.93; 95% CI: 3.51–17.90; *p* < 0.05). The overall effect of the combined category variable was statistically significant in all four models (all *p* < 0.05). In contrast, an elevated ACB score or a higher comorbidity burden in the absence of IADL impairment was not independently associated with recurrent falls (aOR = 1.34; 95% CI: 0.45–4.02; *p* = 0.599 and aOR = 1.27; 95% CI: 0.52–3.14; *p* = 0.601, respectively). None of the covariates included in these models (age, sex, number of medications, CCI, or ACB score category) was independently associated with recurrent falls, except for an ACB score of 2 versus 0 in the two CCI-based models (Table 5).

Interaction analyses. When an interaction term (ACB ≥ 2 × functional impairment) was added to the adjusted ACB-based models, the interaction was not statistically significant for either the Barthel Index (ratio of odds ratios = 0.52; 95% CI: 0.16–1.67; *p* for interaction = 0.271) or the Lawton IADL Scale (ratio of odds ratios = 2.06; 95% CI: 0.55–7.72; *p* for interaction = 0.285). The direction of the interaction estimate differed between the two models, and the confidence intervals were wide. The adjusted odds of fall history associated with ACB ≥ 2 were 3.39 (95% CI: 1.35–8.54) among patients with preserved Barthel Index and 1.75 (95% CI: 0.81–3.79) among those with impaired Barthel Index, and 1.34 (95% CI: 0.45–4.02) among patients with preserved Lawton IADL function and 2.76 (95% CI: 1.27–6.02) among those with impaired IADL function. Accordingly, the combined-category findings are interpreted as joint associations, and no statistical evidence of effect modification was found.

## 4. Discussion

In this retrospective cross-sectional study of older adults, higher anticholinergic burden was associated with a history of recurrent falls; however, functional status showed stronger discriminatory performance than anticholinergic burden alone. Importantly, the combination of impaired instrumental activities of daily living (IADL) and elevated anticholinergic burden was associated with substantially higher odds of a history of recurrent falls. Patients with Lawton IADL scores ≤ 19 and ACB scores ≥ 2 had approximately 12-fold higher odds of recurrent falls compared with those with preserved IADL function and lower anticholinergic burden (OR = 12.04, 95% CI: 5.88–24.66; *p* < 0.05). This combined association remained statistically significant in multivariable analysis after adjustment for age, sex, number of medications, and comorbidity burden (aOR = 7.93, 95% CI: 3.51–17.90; *p* < 0.05), indicating that it was not explained by these factors. However, the formal interaction tests were not significant (*p* for interaction = 0.271 and 0.285), so these findings are best interpreted as a joint association of anticholinergic burden and functional impairment with fall history rather than as evidence that functional status modifies the effect of anticholinergic burden. Importantly, both the Barthel Index and Lawton IADL score remained independently associated with recurrent falls after adjustment for age, sex, anticholinergic burden, medication count, and comorbidity burden. In contrast, individual ACB categories were no longer significantly associated with recurrent falls after adjustment.

Previous studies have reported an association between cumulative anticholinergic burden and falls in older adults [10,20,21]. Wong et al. demonstrated that both polypharmacy and cumulative anticholinergic burden were associated with falls among older adults hospitalized after a fall, and that increasing ACB contributed to fall susceptibility beyond traditional clinical factors such as age and comorbidity burden [10]. Similarly, Salet et al. reported that higher anticholinergic burden, particularly ACB scores ≥ 3, was associated with falls among older adults and suggested a synergistic relationship between anticholinergic burden and depressive symptoms [20]. In another older adult inpatient population, higher ACB scores were associated with dementia, fall risk, and severe disability, supporting the clinical relevance of cumulative anticholinergic exposure in vulnerable older adults [21]. Consistent with these findings, our study demonstrated that ACB scores of 2 and 3 were associated with increased odds of a history of recurrent falls compared with no anticholinergic exposure. Notably, the association was not monotonic: the odds of a history of recurrent falls were higher for an ACB score of 2 (OR = 5.00) than for an ACB score of 3 (OR = 3.07), which does not support a simple dose–response relationship. Because the ACB score was top-coded at 3 (the highest category represents ≥3), the heterogeneity within this category, the small size of the ACB score 2 group (*n* = 27), and the wide, overlapping confidence intervals may partly explain this pattern.

The mechanisms underlying the association between anticholinergic burden and falls are multifactorial. Anticholinergic medications may increase fall risk through central nervous system effects, including sedation, impaired attention, cognitive impairment, and delirium risk, as well as peripheral effects such as blurred vision, orthostatic hypotension, and impaired balance [22,23]. In addition, polypharmacy may contribute to cumulative pharmacological vulnerability, particularly in older adults with reduced physiological reserve. However, polypharmacy and anticholinergic burden are related yet conceptually distinct dimensions of medication exposure. Polypharmacy reflects the quantitative burden of medication use, whereas the ACB score reflects the cumulative anticholinergic properties of the medications used. Therefore, a higher number of medications does not necessarily indicate a higher anticholinergic burden, and anticholinergic burden may be present even in patients taking relatively few medications. Previous evidence has also shown that increasing anticholinergic burden is associated with higher healthcare costs related to falls and fractures, emphasizing the broader clinical impact of medication-related vulnerability [24].

However, the relationship between anticholinergic burden and falls has not been consistently demonstrated across studies. Differences in patient populations, clinical settings, outcome definitions, and anticholinergic burden assessment tools may contribute to heterogeneous findings. For example, Akgün et al. found that anticholinergic medication use categories, as measured by the ACB, were not associated with in-hospital falls among older hospitalized patients [25]. Similarly, Just et al. reported that anticholinergic medications prescribed for overactive bladder were associated with fall-related emergency department visits, whereas cumulative anticholinergic burden was not significantly associated with falls [26]. These findings suggest that anticholinergic burden alone may not fully capture individual susceptibility to falls and that additional patient-level vulnerability factors should be considered.

A major contribution of our study is the description of the joint association of functional status and anticholinergic burden with fall history. Functional measures, particularly the Barthel Index and Lawton IADL Scale, showed higher discriminatory performance than ACB and comorbidity burden. Furthermore, the combined category of functional impairment and elevated anticholinergic burden showed the highest odds of fall history; however, the interaction terms were not statistically significant, and the pattern differed between models (an elevated ACB score alone was associated with fall history among patients with preserved Barthel Index [aOR = 3.39; 95% CI: 1.35–8.54] but not among those with preserved IADL function [aOR = 1.34; 95% CI: 0.45–4.02]). The strongest association was observed in patients with impaired IADL function and elevated ACB scores, which is compatible with, but does not demonstrate, the hypothesis that reduced functional reserve increases vulnerability to medication-related adverse effects. One possible explanation is that older adults with impaired instrumental activities of daily living have greater difficulty compensating for medication-related cognitive, sensory, or motor impairments; this hypothesis requires testing in longitudinal designs.

These findings have important implications for geriatric medication management. Although anticholinergic burden assessment may help identify potentially inappropriate medication exposure, ACB scores should not be interpreted in isolation. A comprehensive geriatric assessment, including evaluation of functional status, may be a more clinically meaningful approach to identifying patients most vulnerable to medication-related falls. In particular, older adults with IADL impairment and elevated anticholinergic burden may be a priority group for medication review and deprescribing interventions.

Psychotropic medications were also more frequently used among patients with recurrent falls in our cohort, particularly SSRIs and antipsychotics. This finding aligns with previous studies linking fall risk-increasing medications to falls among older adults. Ie et al. reported that exposure to multiple fall risk-increasing medication classes was associated with falls among community-dwelling older adults, even among individuals with relatively low medication counts [27]. However, because many psychotropic medications may contribute to anticholinergic burden or sedation, their effects may overlap with broader medication-related vulnerability rather than represent completely independent mechanisms.

This study has several limitations. First, the retrospective cross-sectional design precludes causal conclusions about the relationships among anticholinergic burden, functional impairment, and falls. Reverse causation is possible: falls may lead to functional decline and to changes in prescribing (including the initiation or discontinuation of anticholinergic medications). Functional status may also act as a mediator on the pathway between anticholinergic burden and falls, in which case adjustment for it would constitute over-adjustment, and the adjusted estimates should be interpreted accordingly. Second, the study was conducted in a single older adults population, limiting generalizability to other clinical settings. In particular, our findings may not be directly generalizable to community-dwelling older adults, institutionalized populations, hospitalized patients, or older adults receiving care in other healthcare settings, as differences in functional status, comorbidity burden, medication use, and clinical characteristics may influence the observed associations. Third, fall history was obtained from medical records and may have been affected by recall or documentation bias. Fourth, residual confounding cannot be ruled out despite the evaluation of multiple clinically relevant variables. Although the multivariable models adjusted for several clinically relevant covariates, residual confounding due to unmeasured factors cannot be excluded. Fifth, the cutoffs used to construct the combined categories (Barthel Index ≤ 90, Lawton IADL ≤ 19, ACB ≥ 2, and CCI ≥ 3) were derived from the same sample in which the combined associations were estimated; the combined ORs may therefore be optimistic and require validation in independent cohorts. Sixth, dementia was considerably more frequent among patients with recurrent falls (29.6% vs. 5.9%) and may confound the associations; it was not included in the primary models. In a post hoc analysis additionally adjusted for dementia, the combined categories remained associated with recurrent falls (Barthel Index–ACB model: aOR = 4.95, 95% CI: 2.15–11.39; Lawton IADL–ACB model: aOR = 6.36, 95% CI: 2.75–14.74) and the interaction terms remained non-significant (*p* = 0.278 and *p* = 0.345), although residual confounding by dementia severity cannot be excluded. Seventh, because the ACB score was top-coded at 3, dose–response relationships beyond this level could not be evaluated.

## 5. Conclusions

In older geriatric outpatients, higher anticholinergic burden was associated with recurrent falls in univariable analyses, whereas functional status remained independently associated with recurrent falls after multivariable adjustment. These findings suggest that functional impairment is an important correlate of recurrent falls in older adults, including those with anticholinergic exposure. The combination of impaired instrumental activities of daily living and elevated anticholinergic burden showed the strongest joint association with a history of recurrent falls; however, no statistically significant interaction was observed, and the cross-sectional design precludes conclusions about causality or effect modification. These findings underscore the importance of integrating medication-related risk assessment with functional evaluation in comprehensive geriatric assessment. Evaluating anticholinergic burden alongside functional status may help clinicians recognize older adults with a high burden of fall history who may benefit from targeted medication review, although prospective studies are needed to confirm this.

## Figures and Tables

**Table 1:** Baseline demographic and clinical characteristics of the study population.

Variable	Total (*n* = 347)	Recurrent Falls (−) (*n* = 239)	Recurrent Falls (+) (*n* = 108)	*p* Value
Age, years, median (IQR)	76.0 (70.0–81.0)	74.0 (70.0–79.0)	78.5 (74.0–84.0)	<0.001
Female sex, *n* (%)	235 (67.7)	164 (68.6)	71 (65.7)	0.595
Number of medications, median (IQR)	6.0 (3.0–8.5)	5.0 (3.0–7.5)	7.0 (5.0–10.0)	<0.001
Polypharmacy (≥5 drugs), *n* (%)	222 (64.0)	139 (58.2)	83 (76.9)	0.001
Anticholinergic Cognitive Burden (ACB) score, *n* (%)				
0 point	213 (61.4)	159 (66.5)	54 (50.0)	<0.001
1 point	62 (17.9)	48 (20.1)	14 (13.0)	
2 point	27 (7.8)	10 (4.2)	17 (15.7)	
≥3 point (top-coded)	45 (13.0)	22 (9.2)	23 (21.3)	
Charlson Comorbidity Index, median (IQR)	2.0 (0.0–3.0)	1.0 (0.0–2.0)	2.0 (1.0–4.0)	<0.001
Dementia, *n* (%)	46 (13.3)	14 (5.9)	32 (29.6)	<0.001
Diabetes mellitus, *n* (%)	132 (38.0)	89 (37.2)	43 (39.8)	0.647
Hypertension, *n* (%)	242 (69.7)	164 (68.6)	78 (72.2)	0.499
Stroke history, *n* (%)	28 (8.1)	15 (6.3)	13 (12.0)	0.068
Cardiovascular disease, *n* (%)	97 (28.0)	58 (24.3)	39 (36.1)	0.023
Chronic kidney disease, *n* (%)	99 (28.5)	48 (20.1)	51 (47.2)	<0.001
Psychotropic medication use, *n* (%)	122 (35.2)	68 (28.5)	54 (50.0)	<0.001
Barthel Index, median (IQR)	95.0 (85.0–100.0)	100.0 (90.0–100.0)	85.0 (72.5–95.0)	<0.001
Lawton IADL score, median (IQR)	21.0 (16.0–23.0)	22.0 (19.0–23.0)	16.0 (8.0–21.0)	<0.001
Systolic BP, mmHg, median (IQR)	130.0 (120.0–150.0)	135.0 (120.0–150.0)	130.0 (110.0–140.0)	0.001
Diastolic BP, mmHg, median (IQR)	80.0 (70.0–85.0)	80.0 (70.0–87.5)	75.0 (70.0–81.5)	0.009
Hemoglobin, g/dL, median (IQR)	13.1 (12.0–14.1)	13.1 (12.4–14.1)	12.3 (11.3–13.5)	<0.001
Vitamin D, ng/mL, median (IQR)	25.0 (17.0–34.0)	25.0 (17.0–35.0)	25.0 (17.0–32.0)	0.476
Vitamin B12, pg/mL, median (IQR)	428.0 (287.0–580.5)	428.0 (293.5- 577.0)	423.0 (284.0–697.0)	0.700
TSH, mIU/L, median (IQR)	1.82 (1.21–2.64)	1.85 (1.22–2.55)	1.80 (1.17–2.72)	0.706

Abbreviations: ACB, Anticholinergic Cognitive Burden; BP, blood pressure; IADL, Instrumental Activities of Daily Living; IQR, interquartile range; TSH, thyroid-stimulating hormone.

**Table 2:** Psychotropic medication use.

Variable	Total (*n* = 347)	Fall (−) (*n* = 239)	Fall (+) (*n* = 108)	*p* Value
SSRI use, *n* (%)	83 (23.9)	48 (20.1)	35 (32.4)	0.013
SNRI use, *n* (%)	26 (7.5)	16 (6.7)	10 (9.3)	0.401
Antipsychotic use, *n* (%)	32 (9.2)	11 (4.6)	21 (19.4)	<0.001
Benzodiazepine use, *n* (%)	10 (2.9)	6 (2.5)	4 (3.7)	0.509
Opioid use, *n* (%)	6 (1.7)	3 (1.3)	3 (2.8)	0.380

Abbreviations: SSRI, selective serotonin reuptake inhibitor; SNRI, serotonin–norepinephrine reuptake inhibitor.

**Table 3:** Univariable Logistic Regression Analysis of Factors Associated with Recurrent Falls in Older Adults.

	OR 95% CI	*p*
Age, years, median (IQR)	1.08 (1.04–1.11)	<0.001
Gender; Female, reference	1.14 (0.70–1.84)	0.596
Number of medications	1.13 (1.06–1.20)	<0.001
Polypharmacy	2.38 (1.42–4.00)	0.001
Anticholinergic Cognitive Burden (ACB) score		
0 (reference)	-	
1	0.85 (0.43–1.67)	0.656
2	5.00 (2.16–11.59)	<0.001
≥3 (top-coded)	3.07 (1.58–5.96)	0.001
Charlson Comorbidity	1.30 (1.15–1.46)	<0.001
Dementia	6.76 (3.42–13.35)	<0.001
Diabetes mellitus	1.11 (0.70–1.77)	0.647
Hypertension	1.18 (0.72–1.96)	0.499
Stroke history	2.04 (0.93–4.46)	0.073
Cardiovascular disease	1.76 (1.07–2.88)	0.024
Chronic kidney disease	3.56 (2.17–5.82)	<0.001
Psychotropic medication use	2.51 (1.57–4.02)	<0.001
Barthel Index	0.93 (0.91–0.95)	<0.001
Lawton IADL score	0.87 (0.83–0.90)	<0.001
Systolic BP	0.98 (0.97–0.99)	0.003
Diastolic BP	0.97 (0.95–0.99)	0.014
Hemoglobin	0.75 (0.65–0.87)	<0.001
Vitamin D	0.98 (0.96–1.00)	0.135
Vitamin B12	1.00 (1.00–1.00)	0.079
TSH	0.94 (0.83–1.06)	0.317

Abbreviations: ACB, Anticholinergic Cognitive Burden; BP, blood pressure; CI, confidence interval; IADL, Instrumental Activities of Daily Living; IQR, interquartile range; OR, odds ratio; TSH, thyroid-stimulating hormone. In multivariable analyses, functional status remained independently associated with recurrent falls. In the Barthel-based model, each one-point increase in the Barthel Index was associated with lower odds of recurrent falls (aOR = 0.96, 95% CI: 0.94–0.98; *p* < 0.05). Similarly, in the Lawton-based model, each one-point increase in the Lawton IADL score was independently associated with lower odds of recurrent falls (aOR = 0.91, 95% CI: 0.87–0.96; *p* < 0.05). The overall categorical effect of ACB was statistically significant in both models (*p* < 0.05); however, none of the individual ACB categories differed significantly from ACB score 0 after adjustment (Supplementary Table S1).

**Table 4:** Optimal Cutoff Values of Clinical Scores for Identifying Recurrent Falls Based on the Youden Index.

	Cut Off	AUC	Sensitivity (95% CI)	Specificity (95% CI)	NPV	PPV	Accuracy	Youden Index
Barthel Index	≤90	0.75	63.9 (54.5–72.3)	74.5 (68.6–79.6)	82.0 (76.4–86.6)	53.1 (44.5–61.4)	71.2 (66.2–75.7)	0.38
Lawton (IADL) Scale	≤19	0.74	66.7 (57.3–74.8)	74.1 (68.2–79.2)	83.1 (77.5–87.5)	53.7 (45.3–62.0)	71.8 (66.8–76.2)	0.40
ACB score	≥2	0.61	37.0 (28.5–46.4)	86.6 (81.7–90.4)	75.3 (69.8–80.0)	55.6 (44.1–66.5)	71.2 (66.2–75.7)	0.23
CCI	≥3	0.64	48.1 (39.0–57.5)	76.2 (70.4–81.1)	76.5 (70.7–81.4)	47.7 (38.6–57.0)	67.4 (62.3–72.2)	0.24

Abbreviations: ACB, Anticholinergic Cognitive Burden; AUC, area under the receiver operating characteristic (ROC) curve; CCI, Charlson Comorbidity Index; CI, confidence interval; IADL, Instrumental Activities of Daily Living; NPV, negative predictive value; PPV, positive predictive value. Cutoff values were derived and evaluated in the same sample; performance estimates may therefore be optimistic.

**Table 5:** Association of Combined Functional, Comorbidity, and Anticholinergic Burden Categories with Recurrent Falls.

Combined Model	N Recurrent Falls/N Total (%)	Unadjusted OR (95% CI)	Wald	*p* Value	Adjusted OR (95% CI) ^1^	Adjusted *p* Value
**Model 1: Barthel Index|CCI**						
Barthel Index > 90|CCI < 3 (Ref.)	25/171 (14.6)	-	-		-	
Barthel Index > 90|CCI ≥ 3	14/46 (30.4)	2.55 (1.19–5.45)	5.88	0.015	2.25 (0.98–5.19)	0.056
Barthel Index ≤ 90|CCI < 3	31/67 (46.3)	5.02 (2.65–9.54)	24.40	<0.001	3.37 (1.66–6.87)	<0.001
Barthel Index ≤ 90|CCI ≥ 3	38/63 (60.3)	8.87 (4.59–17.16)	42.12	<0.001	5.01 (2.28–11.01)	<0.001
**Model 2: Barthel Index|ACB score**						
Barthel Index > 90|ACB score < 2 (Ref.)	29/192 (15.1)	-	-		-	
Barthel Index > 90|ACB score ≥ 2	10/25 (40.0)	3.74 (1.53–9.14)	8.41	0.004	3.39 (1.35–8.54)	0.010
Barthel Index ≤ 90|ACB score < 2	39/83 (47.0)	4.98 (2.77–8.94)	28.97	<0.001	3.51 (1.87–6.58)	<0.001
Barthel Index ≤ 90|ACB score ≥ 2	30/47 (63.8)	9.91 (4.85–20.25)	39.65	<0.001	6.15 (2.75–13.77)	<0.001
**Model 3: Lawton IADL Scale|CCI**						
Lawton IADL Scale > 19|CCI < 3 (Ref.)	26/169 (15.4)	-	-		-	
Lawton IADL Scale > 19|CCI ≥ 3	10/44 (22.7)	1.61 (0.71–3.67)	1.32	0.250	1.27 (0.52–3.14)	0.601
Lawton IADL Scale ≤ 19|CCI < 3	30/69 (43.5)	4.23 (2.24–7.97)	19.92	<0.001	2.58 (1.24–5.37)	0.012
Lawton IADL Scale ≤ 19|CCI ≥ 3	42/65 (64.6)	10.04 (5.20–19.39)	47.20	<0.001	5.71 (2.57–12.66)	<0.001
**Model 4: Lawton IADL Scale|ACB score**						
Lawton IADL Scale > 19|ACB score < 2 (Ref.)	31/191 (16.2)	-	-		-	
Lawton IADL Scale > 19|ACB score ≥ 2	5/22 (22.7)	1.51 (0.52–4.42)	0.58	0.444	1.34 (0.45–4.02)	0.599
Lawton IADL Scale ≤ 19|ACB score < 2	37/84 (44.0)	4.06 (2.28–7.23)	22.64	<0.001	2.87 (1.52–5.44)	0.001
Lawton IADL Scale ≤ 19|ACB score ≥ 2	35/50 (70.0)	12.04 (5.88–24.66)	46.30	<0.001	7.93 (3.51–17.90)	<0.001

^1^ Adjusted odds ratios were obtained from multivariable binary logistic regression models. All models were adjusted for age, sex, and number of medications. Models 1 and 3 (CCI-based combined variables) were additionally adjusted for ACB score category (0, 1, 2, and 3; ACB score 0 as reference), and Models 2 and 4 (ACB-based combined variables) were additionally adjusted for the Charlson Comorbidity Index. The overall effect of the combined category variable was statistically significant in all four models (Wald χ^2^ = 18.48, 25.30, 20.77, and 26.69, respectively; all *p* < 0.001, df = 3). The interaction between ACB ≥ 2 and functional impairment, tested by adding a product term to Models 2 and 4, was not significant (*p* for interaction = 0.271 and 0.285, respectively). Abbreviations: ACB, Anticholinergic Cognitive Burden; CCI, Charlson Comorbidity Index; CI, confidence interval; IADL, Instrumental Activities of Daily Living; OR, odds ratio; Ref., reference.

## Data Availability

Data are available from the corresponding author upon reasonable request.

## Supplementary Materials

The following supporting information can be downloaded at: https://www.mdpi.com/article/10.3390/jcm15207811/s1, Table S1: Multivariable Logistic Regression Analyses of Factors Associated with Recurrent Falls.

## Abbreviations

The following abbreviations are used in this manuscript:

ACB	Anticholinergic Cognitive Burden
AUC	Area under the receiver operating characteristic (ROC) curve
BADL	Basic activities of daily living
CCI	Charlson Comorbidity Index
CI	Confidence Interval
IADL	Instrumental Activities of Daily Living
OR	Odds Ratio
SSRIs	Selective serotonin reuptake inhibitors
SNRIs	Serotonin–norepinephrine reuptake inhibitors
