# Supplementary material for: Anticholinergic Burden and Falls in Older Adults: The Role of Functional Status
Source: J Clin Med. 2026 Oct 10;15(20):7811. doi: 10.3390/jcm15207811 (PMC13705503; doi:10.3390/jcm15207811)
Supplement: Supplementary file 1 [file jcm-15-07811-s001.zip › jcm-4491861-supplementary.pdf]

Supplementary Table S1. Multivariable Logistic Regression Analyses of Factors Associated with Recurrent Falls

**Variable**	**Model 1 aOR (95% CI)**	**p value**	**Model 2 aOR (95% CI)**	**p value**
Age	1.03 (0.99–1.07)	0.072	1.03 (0.99–1.07)	0.146
Sex	1.01 (0.58–1.77)	0.949	1.02 (0.58–1.77)	0.934
ACB score 1 vs. 0	0.47 (0.21–1.03)	0.060	0.47 (0.21–1.04)	0.064
ACB score 2 vs. 0	2.04 (0.76–5.41)	0.152	2.11 (0.81–5.49)	0.126
ACB score 3 vs. 0	1.33 (0.59–3.00)	0.478	1.39 (0.62–3.09)	0.415
Number of medications	1.07 (0.99–1.16)	0.086	1.07 (0.99–1.17)	0.069
Charlson Comorbidity Index	1.09 (0.92–1.28)	0.281	1.08 (0.92–1.28)	0.310
Barthel Index	0.96 (0.94–0.98)	<0.001	—	—
Lawton IADL score	—	—	0.91 (0.87–0.96)	<0.001

Model 1: adjusted for age, sex, ACB score category, number of medications, Charlson Comorbidity Index, and Barthel Index.
Model 2: adjusted for age, sex, ACB score category, number of medications, Charlson Comorbidity Index, and Lawton IADL score.
The overall effect of ACB score category was statistically significant in Model 1 (p=0.046) and Model 2 (p=0.040).
